# ReplaceNet: real-time replacement of a biological neural circuit with a hardware-assisted spiking neural network

**DOI:** 10.3389/fnins.2023.1161592

**Published:** 2023-08-10

**Authors:** Sangwoo Hwang, Yujin Hwang, Duhee Kim, Junhee Lee, Han Kyoung Choe, Junghyup Lee, Hongki Kang, Jaeha Kung

**Affiliations:** ^1^Department of Electrical Engineering and Computer Science, DGIST, Daegu, Republic of Korea; ^2^Department of Brain Sciences, DGIST, Daegu, Republic of Korea; ^3^School of Electrical Engineering, Korea University, Seoul, Republic of Korea

**Keywords:** brain-chip interface, dynamic synapses, hardware implementation, spiking neural network, online learning

## Abstract

Recent developments in artificial neural networks and their learning algorithms have enabled new research directions in computer vision, language modeling, and neuroscience. Among various neural network algorithms, spiking neural networks (SNNs) are well-suited for understanding the behavior of biological neural circuits. In this work, we propose to guide the training of a sparse SNN in order to replace a sub-region of a cultured hippocampal network with limited hardware resources. To verify our approach with a realistic experimental setup, we record spikes of cultured hippocampal neurons with a microelectrode array (*in vitro*). The main focus of this work is to dynamically cut unimportant synapses during SNN training on the fly so that the model can be realized on resource-constrained hardware, e.g., implantable devices. To do so, we adopt a simple STDP learning rule to easily select important synapses that impact the quality of spike timing learning. By combining the STDP rule with online supervised learning, we can precisely predict the spike pattern of the cultured network in real-time. The reduction in the model complexity, i.e., the reduced number of connections, significantly reduces the required hardware resources, which is crucial in developing an implantable chip for the treatment of neurological disorders. In addition to the new learning algorithm, we prototype a sparse SNN hardware on a small FPGA with pipelined execution and parallel computing to verify the possibility of real-time replacement. As a result, we can replace a sub-region of the biological neural circuit within 22 μs using 2.5 × fewer hardware resources, i.e., by allowing 80% sparsity in the SNN model, compared to the fully-connected SNN model. With energy-efficient algorithms and hardware, this work presents an essential step toward real-time neuroprosthetic computation.

## 1. Introduction

In the field of systems neuroscience, studies on brain-machine interface (BMI) to replace semi-permanent functions of the human brain have been conducted for the treatment of neurological disorders or the use of neuroprosthetics (Zhang et al., 2020). For example, Song et al. (2007) have replaced the function of damaged hippocampal neurons with a mathematical model. The model predicts the electrical transmission between neurons so that similar electrical functionality can be artificially generated for damaged neurons. Recently, the authors in Hampson et al. (2018) have demonstrated that electrical stimulation to the biological neuron improves memory function in human subjects by predicting electrical transmission between neurons. However, most studies on BMI are based on traditional offline learning, making it challenging to actively cope with biological learning such as neuroplasticity. Moreover, the complexity of a mathematical model becomes intractable as a biological neural circuit to be replaced becomes larger (Song et al., 2016; She et al., 2022).

Recently, artificial neural networks (ANNs) were used in explaining how the brain learns to perform perceptual and cognitive tasks (Richards et al., 2019). Specifically, brain-inspired spiking neural networks (SNNs) were utilized to understand activity patterns of neural circuits (Doborjeh et al., 2019; Lee et al., 2019; Kumarasinghe et al., 2021). Several recent studies have shown promising results on the capability of understanding a high-level brain functionality using SNN models, e.g., decoding neuro-muscular relationships (Kumarasinghe et al., 2021) or establishing a peripheral nervous system (Lee et al., 2019). Owing to the biological interpretability of the SNN model, it is even possible to mimic the microscopic behaviors of neural circuits, i.e., spike timings, firing rates, and burst patterns (Sun et al., 2010; Dominguez-Morales et al., 2021). In addition to biological plausibility, SNNs are energy efficient because they only compute when spikes are present (*event-driven*). Therefore, many studies have focused on improving the training accuracy of SNNs by introducing surrogate gradient descent (Fang et al., 2021; Zheng et al., 2021) or converting pre-trained ANNs into SNNs (Han and Roy, 2020; Han et al., 2020) even for tasks that are mainly used for ANNs such as computer vision.

In addition to the algorithmic improvement, neuromorphic hardware chips have been designed, either analog (Benjamin et al., 2014) or digital (Akopyan et al., 2015; Davies et al., 2018), to process large-scale asynchronous SNNs efficiently. The main objective of neuromorphic hardware is to simulate the behavior of a large number of neurons in real-time with low power consumption. However, prior works suffer from the inability to support, or partially support, biologically plausible neuron models, or synaptic learning rules. To address these challenges, Lee et al. (2018) and Baek et al. (2019) have presented programmable SNN hardware that supports a wide range of neuron models and synaptic learning rules. Another approach is to use an FPGA platform, which allows flexible modification of neuron models and network structures by reconfiguring the hardware architecture (Cheung et al., 2016; Sripad et al., 2018). To efficiently process large-scale SNNs on multiple FPGA chips, SNN hardware with novel routing algorithms for energy-efficient computation of nonlinear neuron models have been proposed (Yang et al., 2019). Moreover, efficient implementations and algorithms have been proposed to support the mechanisms of various biological brain regions, such as the cerebellum and hippocampus, in large-scale SNNs (Yang et al., 2021a,b).

In short, SNNs can imitate biological neural networks (BNNs) more closely than other ANN counterparts with higher energy efficiency. Therefore, SNN is an ideal option in neuroprosthetics modeling to increase energy efficiency and biological plausibility (Li et al., 2021). In order to predict precise spike timings, several supervised learning rules have been proposed (Wang et al., 2020), and are typically divided into gradient descent learning (Bohte et al., 2002) and STDP-based learning (Ponulak and Kasiński, 2010). Although gradient descent learning can solve complex tasks, it is unsuitable for online learning because of its higher parameter dependence and slower learning speed than the synaptic plasticity learning (Lobo et al., 2020). STDP-based supervised learning is more suitable for online learning. To minimize the complexity of STDP-based supervised learning, we present a simple yet effective learning method called STDP-assisted spike-timing learning (SA-STL). With the help of our SA-STL rule, we can aggressively remove less important synapses dynamically in the SNN model with a little loss in the learning capability.

In this work, we focus on reproducing the target spike train with a limited number of synapses in the SNN model. It is validated using both *synthetic data* and *our cell culture data*. Then, this paper provides an initial set of experiments to understand the possibility of replacing a sub-region of a neural circuit by training a recurrent SNN. To directly replace the sub-region of the neural circuit, we map each artificial neuron in our SNN model to each cultured biological neuron being monitored by a single probe in a microelectrode array (MEA). Connectivity between artificial neurons is trained by STDP-assisted supervised learning to generate a spike train that is identical to the desired spike train of the MEA. To demonstrate the real-time replacement, we implemented our SNN model on a hardware platform, i.e., Xilinx PYNQ-Z2 board, running at 50 MHz with pipelined execution. Overall, the key contributions of this work can be summarized as:

1. **Dataset Collection**: We cultured a hippocampal neuronal network to collect spike activities of biological neurons for more realistic experiments. The data is collected every 12 h over 10 days, which provides 20 sessions in total.
2. **Learning Algorithm**: We replaced the sub-region of the biological neural network by predicting spikes based on input spikes through an online STDP-based supervised learning rule. We proposed a novel learning method that reliably removes synapses in the SNN model, which leads to a more efficient hardware implementation. This results in the hardware design occupying less area and consuming less power.
3. **Hardware Implementation**: We implemented a sparse SNN hardware on FPGA that predicts spikes of biological neurons in the replaced region in real-time (i.e., < 1 ms).

The remainder of this paper is organized as follows. Section 2.1 introduces various neuron models and synaptic learning rules. Section 2.2 presents our SA-STL rule that dynamically selects important synapses to be connected when training an SNN model. In Section 2.3, we provide an experimental setup for replacing a sub-region of a neural circuit with the trained SNN model. Section 3.2 presents the details of SNN hardware architecture and analyzes the spike prediction accuracy using the actual hardware for real-time replacement. Then, we conclude the paper in Section 4.

## 2. Materials and methods

### 2.1. Preliminaries: learning precise spike timings

#### 2.1.1. Spiking neuron models

A biological neuron's membrane potential is defined by the difference between the extra- and intra-cellular potentials due to ion concentration gradients. The neuron's membrane potential increases by external stimuli (depolarization). The spike propagates to other post-synaptic neurons after the cell membrane potential depolarizes to its threshold level. As a spike generate, the membrane potential decreases (repolarization) until it reaches the resting state. At the resting state, the membrane potential settles to resting membrane potential, e.g., *E*_*rest*_= −70 mV, and is stable during the refractory period, e.g., *T*_*r*_= 2 ms. In its refractory period, the neuron cannot generate any spikes. This complicated process of neuronal behavior has been modeled and imitated by an artificial neuron, i.e., leaky integrate-and-fire (LIF), quadratic integrate-and-fire (QIF), depending on the artificial neuron model, the computational complexity varies regarding membrane decay, spike accumulation, spike initiation, and refractory behavior (Lee et al., 2018).

Since our goal is to mimic BNNs by SNNs in the real-time and energy-constrained environment, we stick to a relatively simple LIF model throughout the paper. The dynamics of the LIF neuron model is defined as


(1) $$\begin{array}{l} \begin{array}{cc} \tau_{j}\frac{dv_{j}\left(t\right)}{dt} & =\left(E_{rest}-v_{j}\left(t\right)\right)+\overset{N_{pre}}{\underset{i=1}{\sum }}w_{ij}\delta_{i}\left(t-t^{i}\right)\\ \;v_{j}\left(t\right) & =E_{rest}\text{when}\;v_{j}\left(t\right)>V_{\theta }, \end{array} \end{array}$$

where *i* or *j* is the index of a pre-or post-synaptic neuron, *N*_*pre*_ is the number of pre-synaptic neurons, *v*_*j*_ is the membrane potential of the post-synaptic neuron *j* (negative value), and τ_*j*_ is the time constant of the membrane potential. The *w*_*ij*_ is the strength of a synaptic connection between the neuron *i* and *j*, *t*^*i*^ is the spike time at the pre-synaptic neuron *i*, and δ_*i*_(·) is the Dirac delta function, i.e., δ(*x*) = 1 (if *x* = 0) or 0 (otherwise). Each pre-synaptic neuron has synapses that convey a weighted spike to the post-synaptic neuron increasing *v*_*j*_. The synaptic strength determines the amount of change in the membrane potential of the post-synaptic neuron. When the *v*_*j*_ reaches the pre-determined threshold *V*_θ_, the neuron *j* generates the spike, and its membrane potential resets to *E*_*rest*_. When there are no stimuli to the post-synaptic neuron, the membrane potential constantly falls over time, which is determined by the term “*E*_*rest*_−*v*_*j*_(*t*).” Our work was performed with *V*_θ_ fixed at −55 mV and τ_*j*_ at 10 ms.

#### 2.1.2. Synaptic learning rules

The dynamics of a neuron in Equation (1) involves *w*_*ij*_ which represents the strength of the synaptic connection between the neuron *i* and *j*. This synaptic strength determines the amount of change in the membrane potential of the post-synaptic neuron. When constructing an SNN model, the weight update rule, the so-called synaptic learning rule, becomes essential to estimate the spike timings of post-synaptic neurons precisely. Therefore, various synaptic learning rules were studied in the field of computational neuroscience (Markram et al., 1997; Bi and Poo, 1998; Pfister and Gerstner, 2006).

##### 2.1.2.1. Spike-timing-dependent plasticity (STDP)

The most common and unsupervised synaptic learning rule is the STDP rule. Following the standard STDP rule, each weight *w*_*ij*_ is potentiated or depressed by the relative time difference between the pre-synaptic and post-synaptic spikes. The pair-wise STDP rule is defined as:


(2) $$\begin{array}{l} \Delta w_{ij}=A_{+}x_{i}\left(t\right)\cdot \delta \left(t-t^{j}\right)-A_{-}x_{j}\left(t\right)\cdot \delta \left(t-t^{i}\right), \end{array}$$

where *t*^*i*^ or *t*^*j*^ is the spike time of the neuron *i* or *j*, *A*_+_ (or *A*_−_) is the coefficient for the weight potentiation (or depression), and *x*_*i*_(*t*) or *x*_*j*_(*t*) is the trace of the neuron *i* or *j*. The trace of each neuron is used to determine the amount of increase/decrease in its membrane potential depending on how close the pre-synaptic spikes and post-synaptic spikes are (Pfister and Gerstner, 2006). The trace of the pre-synaptic neuron *i*, i.e., *x*_*i*_(*t*), may contain the history of all spikes at previous time steps, i.e., all-to-all interactions. Another type of the trace model considers only the most recent spike, i.e., nearest-neighbor interactions. Since our experimental results showed little difference between the two, we update the trace of each neuron with all-to-all interactions, which is defined as


(3) $$\begin{array}{l} \frac{dx_{i}\left(t\right)}{dt}=-\frac{x_{i}\left(t\right)}{\tau_{x}}+\delta \left(t-t^{i}\right), \end{array}$$

where τ_*x*_ is the time constant of the trace. However, the objective of the STDP rule is not to learn precise spike timings at the post-synaptic neuron. Instead, it focuses on identifying how strong/weak each synaptic connection is by looking at every pre-and post-spike pair.

##### 2.1.2.2. Remote supervised method (ReSuMe)

To train synaptic weights so that neurons fire spikes at desired time steps, STDP-based supervised learning rules have been proposed (Ponulak and Kasiński, 2010; Mohemmed et al., 2013; Xu et al., 2013b; Yu et al., 2013; Zhang et al., 2017, 2018). The main difference between supervised learning and STDP rules is that supervised methods quantify spike timing errors to precisely predict the desired spike timings. ReSuMe (Ponulak and Kasiński, 2010) is a supervised learning rule based on the Widrow-Hoff rule, i.e., the compound of two Hebbian processes (Kistler, 2002; Roberts and Bell, 2002). ReSuMe uses both desired spikes (target) and output spikes that the SNN model incurs. ReSuMe can be interpreted as an STDP-like process relating the pre-synaptic spikes [as a trace *x*_*i*_(*t*); Equation 3] with the timing error [$S_{j}^{d}\left(t\right)-S_{j}^{o}\left(t\right)$], which is defined as


(4) $$\begin{array}{l} \Delta w_{ij}=\left(S_{j}^{d}\left(t\right)-S_{j}^{o}\left(t\right)\right)\left(a_{d}+x_{i}\left(t\right)\right), \end{array}$$

where *w*_*ij*_ is a synaptic weight from a pre-synaptic neuron *i* to a post-synaptic neuron *j*, *a*^*d*^ is a constant for setting a specific firing rate, $S_{j}^{d}$ is the desired spike train at the target neuron *j*, and $S_{j}^{o}\left(t\right)$ is the output spike train from the corresponding spiking neuron *j*. Here, the spiking neuron represents an artificial neuron in the SNN model. The spike train of a neuron can be expressed as


(5) $$\begin{array}{l} S_{j}\left(t\right)=\underset{f}{\sum }\delta \left(t-t_{j}^{f}\right), \end{array}$$

where $t_{j}^{f}$ is the spike time of the *f*th spike at the neuron *j*.

##### 2.1.2.3. Supervised learning with a kernel function

Other STDP-based supervised synaptic learning rules try to transform discrete spike trains to continuous-valued trains with a kernel function κ(*t*) (Mohemmed et al., 2013; Yu et al., 2013). In Spike Pattern Association Neuron (SPAN) method (Mohemmed et al., 2013), the authors convolve all spike trains, i.e., input, output, and desired spike trains, with an alpha kernel so that gradient descent can be used to minimize the timing error. Then, the spike timing error is defined as the difference between (transformed) desired and output spike trains. The synaptic learning rule of SPAN can be expressed as


(6) $$\begin{array}{l} \begin{array}{c} \Delta w_{ij}=\left(\underset{t_{d}^{f}<t}{\sum }\kappa \left(t-t_{d}^{f}\right)-\underset{t_{o}^{f}<t}{\sum }\kappa \left(t-t_{o}^{f}\right)\right)\underset{t_{i}^{f}<t}{\sum }\kappa \left(t-t_{i}^{f}\right),\\ \kappa \left(t-t_{i}^{f}\right)=\frac{e}{\tau }\left(t-t_{i}^{f}\right)e^{\frac{-\left(t-t_{i}^{f}\right)}{\tau }}, \end{array} \end{array}$$

where $t_{i}^{f}$ is the spike timing at a pre-synaptic neuron, $t_{d}^{f}$ (or $t_{o}^{f}$) is the desired (or output) spike timing at a post-synaptic neuron, and τ is the decay constant. Instead of convolving all the spike trains, Precise Spike-Driven plasticity rule (PSD; Yu et al., 2013) only convolves the input spike train with a kernel function having two independent decay constants. The synaptic learning rule of PSD can be expressed as


(7) $$\begin{array}{l} \begin{array}{c} \Delta w_{ij}=\left(S_{j}^{d}\left(t\right)-S_{j}^{o}\left(t\right)\right)\underset{t_{i}^{f}<t}{\sum }\kappa \left(t-t_{i}^{f}\right),\\ \kappa \left(t-t_{i}^{f}\right)=V_{0}\left(e^{\frac{-\left(t-t_{i}^{f}\right)}{\tau_{s}}}-e^{\frac{-\left(t-t_{i}^{f}\right)}{\tau_{f}}}\right), \end{array} \end{array}$$

where $t_{i}^{f}$ is the spike timing of a pre-synaptic neuron, *V*_0_ is the normalization factor, τ_*s*_ is the slow decay constant, and τ_*f*_ is the fast decay constant. The ratio τ_*s*_/τ_*f*_ is set to 4.

### 2.2. Precise spike-timing learning with STDP-assisted functional connectivity estimation

**Figure 1:**
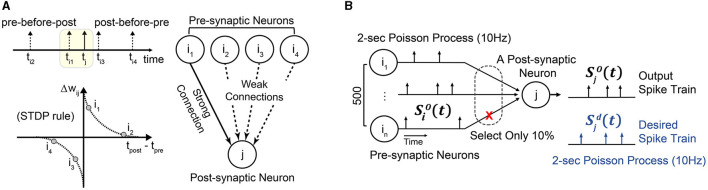
**(A)** A simple illustration of STDP-assisted functional connectivity estimation used in the proposed SA-STL learning rule. **(B)** A simple experimental setup to validate the effectiveness of the proposed SA-STL learning rule. It consists of 500 pre-synaptic neurons connected to a single post-synaptic neuron. The firing rate of both pre- and post-synaptic neurons is set to 10 Hz.

This section proposes a simple yet effective learning rule, STDP-assisted spike-timing learning (SA-STL), that accurately predicts precise spike timings with limited synapses between neurons. Since we target real-time processing on hardware, saving the memory footprint and computing resources is crucial. It can be done by dynamically estimating the useful connections within the target neural network by using a simple STDP learning rule (Figure 1A). To demonstrate the effectiveness of the proposed SA-STL, we generated synthetic data consisting of 500 pre-synaptic neurons and a single post-synaptic neuron. The objective is to precisely predict the spike timings at the post-synaptic neuron with an SNN model.

#### 2.2.1. Learning precise spike timings with synthetic data

In our synthetic data, the spike train for each neuron is a 2 s Poisson process with a firing rate of 10 Hz (Figure 1B). We have set the firing rate of pre-and post-synaptic neurons to 10 Hz, which is lower than the other work (Mohemmed et al., 2013; Yu et al., 2013; Zhang et al., 2018), to mimic the behavior of hippocampal neural networks. Then, we can use any supervised learning rules, e.g., ReSuMe, PSD, and SPAN, to precisely predict the spike timings at the post-synaptic neuron [defined as the desired spike train $S_{j}^{d}\left(t\right)$]. In our simulation, each synaptic weight *w*_*ij*_ that connects the pre-synaptic neuron “*i*” to the post-synaptic neuron “*j*” is trained for 100 epochs. When the training is completed, output spike train $S_{j}^{o}\left(t\right)$ of the SNN model trained by the selected learning rule should fire spikes simultaneously as $S_{j}^{d}\left(t\right)$. To reduce the complexity of the SNN model, our SA-STL learning keeps the important synapses, e.g., only 10%, and cuts off the rest. Here, the important synapses help the SNN model improve the accuracy of the spike timing prediction.

#### 2.2.2. Relation between STDP and STDP-based supervised learning rules

In order to cut the synapses that have little impact in predicting the precise timings of desired spike trains, we need a simple metric that can determine the importance of each synapse on the fly during training. As Section 2.1.2.1 explains, the STDP rule only focuses on the relative timing between pre- and post-synaptic spike pairs. If the post-synaptic spike follows the pre-synaptic spike (pre-before-post), the weight *w*_*ij*_ is potentiated. On the contrary, if the post-synaptic spike comes before the pre-synaptic spike (post-before-pre), the weight *w*_*ij*_ is depressed. After the STDP learning, the synapse can be classified as excitatory (or inhibitory) if the net weight change is positive (or negative). However, none of the previous studies have shown how excitatory or inhibitory synapses, determined by the STDP rule, affect the accuracy of spike timing predictions. Thus, we extracted the relation between the weight change learned by the STDP rule and the trained weights via supervised learning rules, such as ReSuMe.

**Figure 2:**
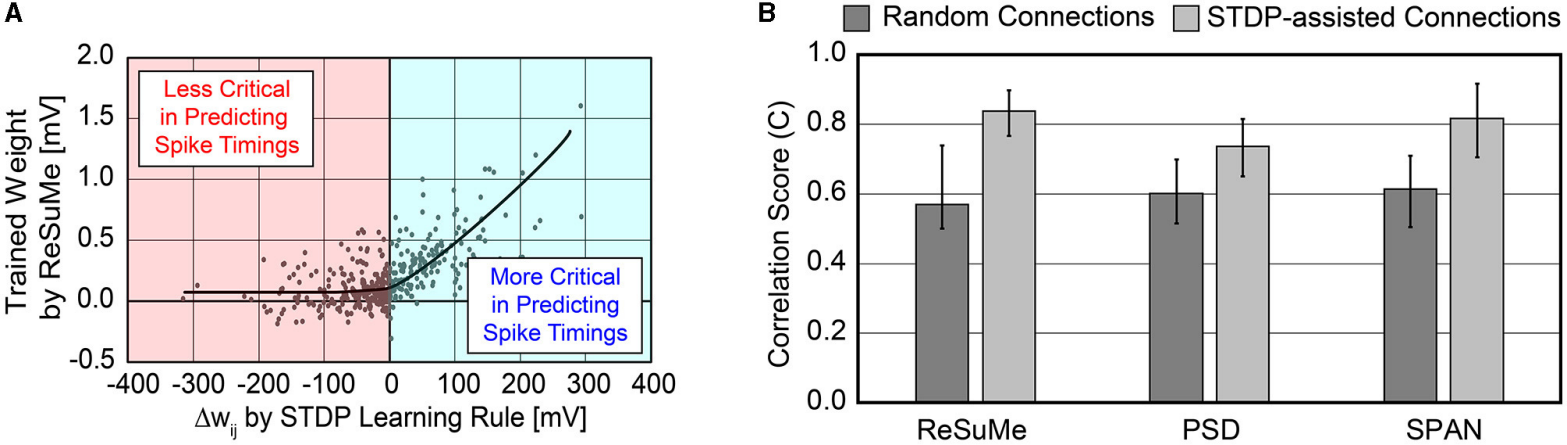
**(A)** Relation between weight changes (Δ*w*_*ij*_) by STDP learning rule and trained weight values by ReSuMe learning rule on all 500 synaptic connections. The ReSuMe focuses on predicting the precise spike timings. **(B)** Comparison of the accuracy between randomly connected SNNs and SNNs using the proposed SA-STL rule. Only 10% of synapses out of 500 are kept for both cases.

As shown in Figure 2A, the magnitude of the trained weights via ReSuMe has a high correlation with the weight change computed by the STDP rule when Δ*w*_*ij*_>0. This implies that excitatory synapses with large Δ*w*_*ij*_, i.e., strong “pre-before-post” connections, can be considered necessary in predicting the spike timings. In other words, disconnecting these strong connections can devastate the accuracy of reproducing the desired spike train $S_{j}^{d}\left(t\right)$. The trained weights via ReSuMe are clustered near 0 mV for those synapses classified as inhibitory by the STDP rule. It implies that the inhibitory synapses can be safely disconnected when predicting the spike timings with a limited number of synapses. This trend was also observed using other supervised learning methods, such as PSD and SPAN. Based on this analysis, we selected the STDP rule for dynamically pruning insignificant synapses during the precise spike-timing learning to achieve higher hardware efficiency.

#### 2.2.3. Prediction accuracy on synthetic data

To validate the effectiveness of the proposed SA-STL, we analyzed the accuracy of predicting the desired spike train $S_{j}^{d}\left(t\right)$ by keeping only 10% of synapses out of 500. The proposed SA-STL rule is summarized in Algorithm 1. As a spike-timing learning rule used in line 11 of Algorithm 1, we selected a supervised learning rule presented in Sections 2.1.2.2 and 2.1.2.3, i.e., ReSuMe, PSD, or SPAN. For every session, SA-STL runs two separate learning rules: (i) the supervised learning rule that trains the weights to generate the desired spike train precisely, and (ii) the STDP rule for estimating functional connectivity is used to select useful connections in the next training session. For the experiment using the synthetic data, input, and desired spike patterns [*S*_*i*_(*t*) and $S_{j}^{d}\left(t\right)$] are fixed over training sessions. One can consider each session as a training epoch. Thus, the evaluation of functional connectivity within an SNN model happens only in the first session. After the first session, only a subset of pre-synaptic neurons is connected to a post-synaptic neuron *j*, and the selected supervised learning rule trains weights.

**Algorithm 1:**
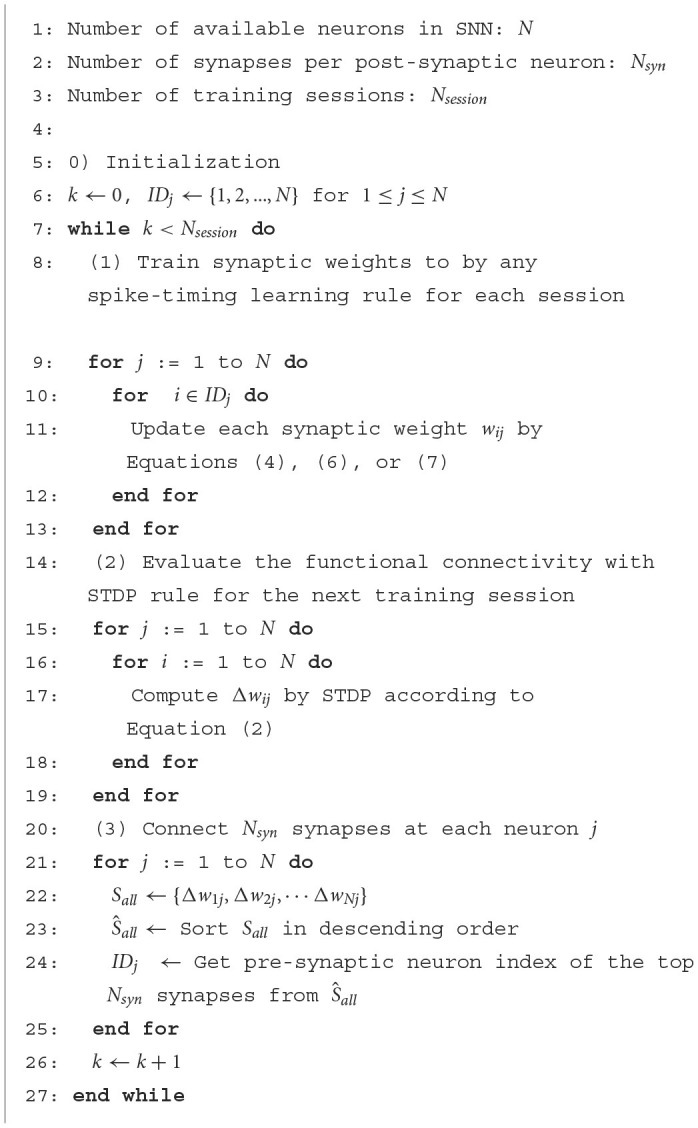
STDP-assisted spike-timing learning (SA-STL).

**Figure 3:**
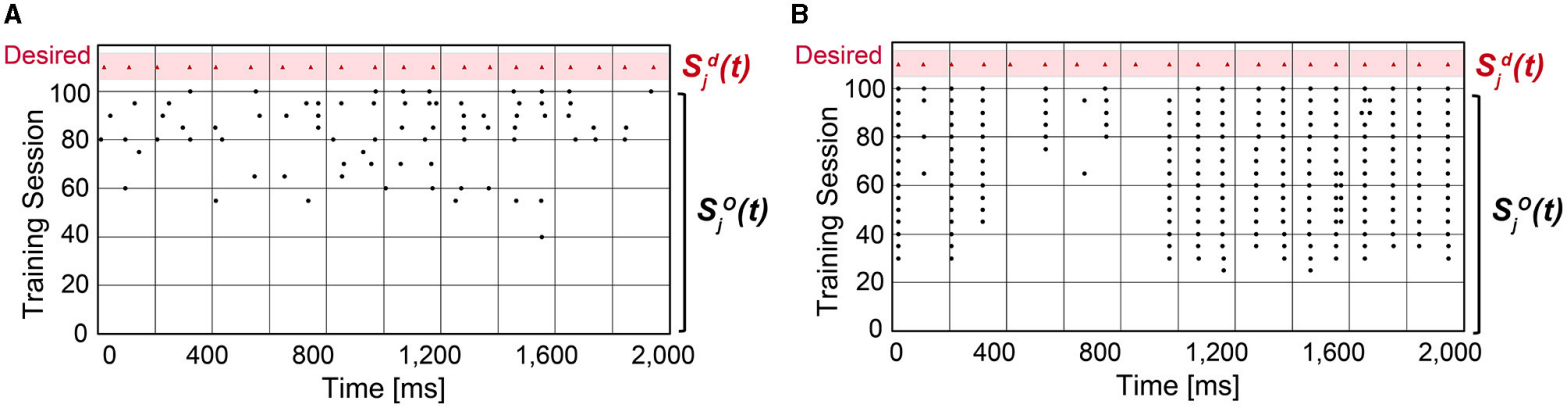
Raster plot of the output spike train $S_{j}^{o}\left(t\right)$ from an SNN model compared with the desired spike train $S_{j}^{d}\left(t\right)$. **(A)** An SNN model in which 50 pre-synaptic neurons are randomly selected that connect to a post-synaptic neuron *j*. **(B)** An SNN model in which 50 pre-synaptic neurons are selected by running the STDP rule.

We compared the accuracy of spike-timing learning when 10% of synapses were randomly selected and when selected by the STDP rule. The accuracy is measured by a correlation-based metric presented in Schreiber et al. (2003). The correlation-based score is one of the conventional methods to evaluate the accuracy of predicting the desired spike train, and it can be expressed as


(8) $$\begin{array}{l} C=\frac{\vec{s^{d}}\cdot \vec{s^{o}}}{\left|\vec{s^{d}}||\vec{s^{o}}\right|}, \end{array}$$

where $\vec{s^{d}}$ (or $\vec{s^{o}}$) is the desired (or output) spike train that is Gaussian filtered, and $\left|\vec{s}\right|$ is the Euclidean norm of $\vec{s}$. As *C* gets closer to 1, the confidence in predicting the desired spike train is higher. When using SA-STL, we select the top 10% synapses with large Δ*w*_*ij*_ obtained by the STDP rule (*N*_*syn*_ = 50). Figure 2B compares the prediction accuracy in terms of “*C*” provided in Equation (8). Experiments were performed for 20 trials, and the SA-STL approach achieves an accuracy improvement by 15–27% when the SNN model allows only a tiny fraction of synapses (10% in this simulation). Note that the best accuracy of the randomly connected SNN model fails to exceed the mean accuracy of SA-STL in all test cases. This accuracy improvement with a limited number of synapses is more evident by looking at the raster plot of both desired and output spike train in Figure 3. The SNN model that randomly connects pre-synaptic neurons missed 55% of the target spikes (20 spikes in total) even after the training with 100 sessions. With the SA-STL rule, the training converges much faster, and 20% of the target spikes are missed after the training.

**Figure 4:**
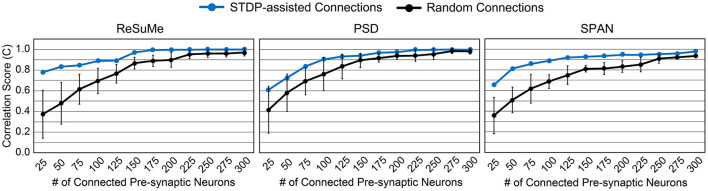
Accuracy comparison by varying the number of connected pre-synaptic neurons. Three different supervised learning rules, i.e., ReSuMe, PSD, and SPAN, are tested to validate the proposed SA-STL rule.

We can expect a higher prediction accuracy by allowing more connections within the SNN model. Figure 4 shows how the accuracy improves as more synapses are connected in the SNN model. By connecting more than 300 pre-synaptic neurons, i.e., over 60%, the desired spike train was perfectly reproduced (*C*≃1). The smaller the number of synapses, the greater the accuracy gap between the randomly connected SNN model and the STDP-assisted SNN model. In addition, among the three supervised learning rules, ReSuMe shows the best accuracy when the number of synapses in the SNN is small (< 100). The effectiveness of SA-STL increases with larger models. To verify this, we have generated synthetic data with 50,000 pre-synaptic neurons. First, we assumed 0.1% of the pre-synaptic neurons, i.e., 50 neurons, are connected to the output neuron. Then, the spike prediction accuracy of 33.7% on average is observed with random connections, while 91.0% is achieved with the proposed SA-STL. If more than 1% of the pre-synaptic neurons in a randomized experiment are connected, the prediction accuracy increases to 80.0% on average but varies widely depending on the connectivity pattern. Therefore, the SA-STL method provides more reliable training as the model size increases. This set of experiments shows that connections in the SNN model can be reliably initialized and re-connected using the STDP rule during the precise spike-timing learning.

### 2.3. Replacement of a biological neural circuit

As mentioned in Section 1, the main goal of precisely estimating spike timings in this work is to replace a sub-region of a biological neural circuit with an SNN model. To physically replace the part of the neural circuit, we eventually need an extremely small SNN hardware. We utilize the proposed SA-STL rule to maintain high learning capability while reducing the SNN model complexity. The reduction in the model complexity leads to a more efficient hardware implementation, as discussed in Section 3.2.1.

#### 2.3.1. Multichannel recording experimental setup and cell culture data collection

To collect biologically meaningful neural recording data, we cultured embryonic hippocampal neurons on a microelectrode array (MEA) chip (60MEA200/30iR-ITO-gr, Multi-Channel Systems MCS GmbH, Germany) in which we have 60 electrodes for each single-cell neural recording. The hippocampus in the brain is in charge of memory storage and reminding memory. Also, the embryonic phase typically shows noticeable brain development and differentiation. Thus, cell-to-cell signal transmission is actively generated during embryonic development, which is one of the reasons why we chose hippocampal neurons extracted from rat embryos. Despite the lack of sensory inputs or motor outputs of the cultured neuronal network, it has been known that the cultured network still contains electrophysiological signal patterns similar to the brain *in vivo* (Belle et al., 2018). In addition, the *in vitro* cultured network can be maintained for a long time, e.g., more than 1 or 2 months. Thus, it gives a great experimental biological model for us to develop an SNN model for training and replacing the part of a cultured neural circuit.

We incubated the cultured hippocampal neurons (1,000 *cells*/*mm*^2^) on the MEA chips at (37°C, 5% CO_2_) for long-term stability while we recorded the neuronal activity signals over several days. We chose cell density to guarantee enough cell-to-cell interactions in the network while avoiding overcrowded cells for reduced network stability. We used a 60-channel pre-amplifier headstage (MEA2100-Mini-HS60) in the humid incubator. Spontaneous extracellular neural spikes were recorded at 25 kHz sampling frequency and digitized at 24-bit data resolution. The recorded multi-channel signals were obtained after digital bandpass filtering (from 200 Hz to 3.5 kHz with the 2nd order Butterworth filter). In order to use the recording data as inputs to the SNN model, only the timestamps of the recorded extracellular neural spikes were used after a conventional threshold-based spike detection method. Each spike recording session is 10 min long, which becomes one training session in Algorithm 1, and the periodic recording was conducted every 12 h over 10 days without any physical movement. The recording was initiated at least after 14 days *in vitro* (DIV) to allow complete synapse connections in the neuronal network. There are 20 sessions in total prepared for experiments from one culture model on replacing a biological neural circuit in the following sections. Please find more detailed experimental procedures in the Supplementary material.

##### 2.3.1.1. Ethics approval statement

All experiments were performed in accordance with the guidance of the Institutional Animal Care and Use Committee (IACUC) of Daegu Gyeongbuk Institute of Science and Technology (DGIST), and all experimental protocols were approved by IACUC of DGIST (DGIST-IACUC-21041903-0002).

#### 2.3.2. Problem definition and SNN structure

**Figure 5:**
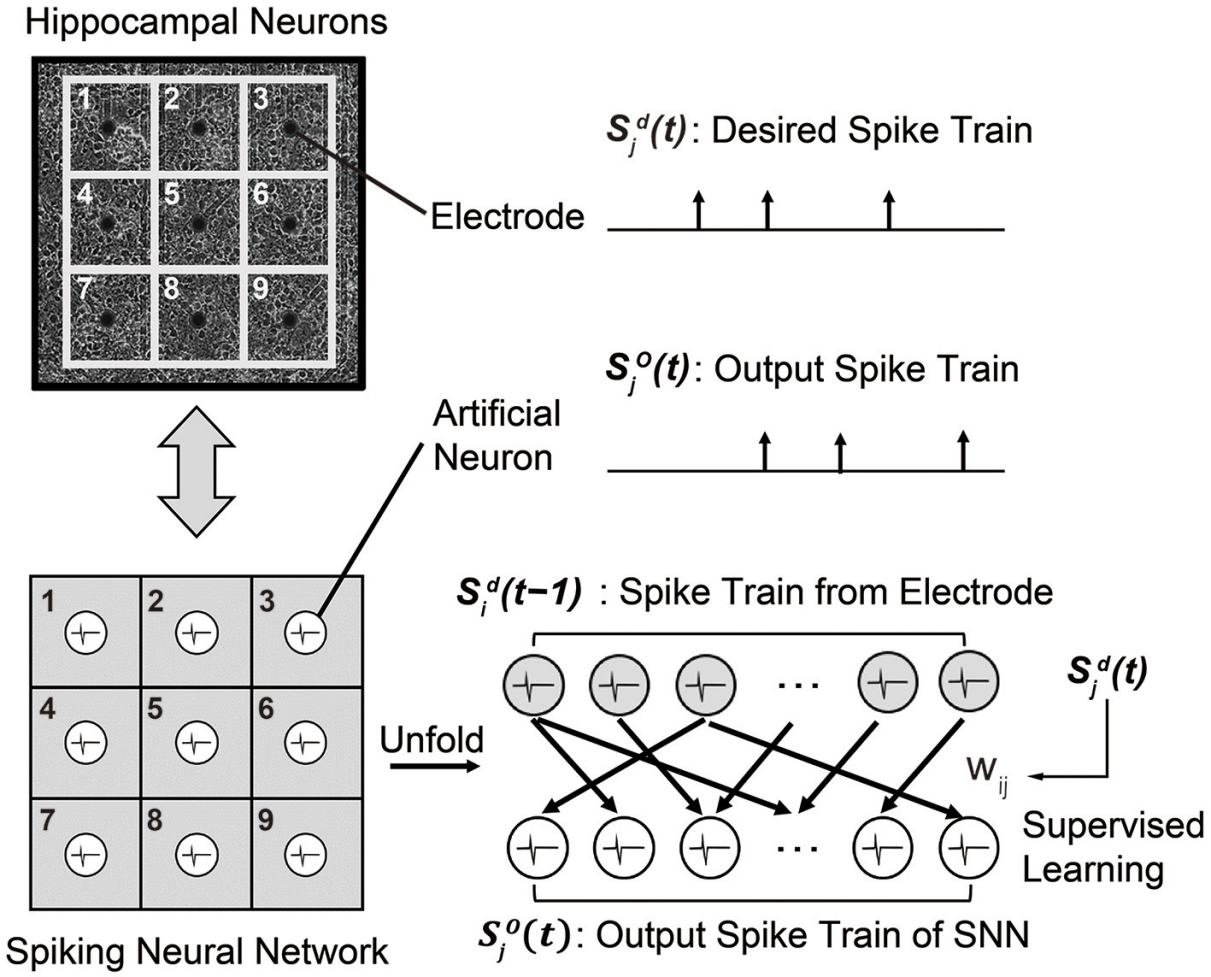
A recurrent SNN model that is designed to mimic the behavior of a biological neural circuit. Each synaptic weight is trained by the supervised learning rule so that a spike train $S_{j}^{o}\left(t\right)$ from the SNN model becomes identical to the desired spike train $S_{j}^{d}\left(t\right)$ of the biological neural circuit.

To replace a sub-region of cultured hippocampal networks, i.e., biological neural circuits, we designed a recurrent SNN composed of artificial spiking neurons. As shown in Figure 5, the SNN model has the same number of neurons as the number of electrodes in the MEA, i.e., 60 in our experiments. Then, the objective is to train the SNN model to generate a spike train $S_{j}^{o}\left(t\right)$ that is identical to the desired spike train $S_{j}^{d}\left(t\right)$ of the MEA. Here, an artificial neuron index *j* represents the paired electrode in the MEA. As a neuron model, we use the simplest LIF model presented in Section 2.1.1 to realize real-time spike prediction on hardware. At each time step (1 ms), the membrane potential of each neuron *j* is updated according to the pre-synaptic spikes that are fired from the previous time step [$S_{i}^{d}\left(t-1\right)$], as we are using the recurrent SNN model. The index *i* represents pre-synaptic neurons connected to the neuron *j*. If the membrane potential of the neuron *j* exceeds the pre-determined threshold, it fires a spike which becomes $S_{j}^{o}\left(t\right)$.

To train the SNN model capable of replacing a sub-region of the biological neural circuit, we use the measured spike train of the MEA as the target spike train $S_{j}^{d}\left(t\right)$. Then, we use one of the supervised learning rules presented in Section 2.1.2 so that the output spike train $S_{j}^{o}\left(t\right)$ of the SNN matches $S_{j}^{d}\left(t\right)$. The supervised learning rules are based on the following rules: (i) a synaptic weight *w*_*ij*_ is decreased when the neuron *j* fires at undesired time, and (ii) the *w*_*ij*_ is increased when the neuron *j* fires at desired time. Since these training methods are event-driven, the number of computations is less than that of other training methods, such as gradient-based algorithms (Bohte et al., 2002; Xu et al., 2013a). The input spike train $S_{i}^{d}\left(t-1\right)$ is the measured spike train of the MEA at the previous time step. The time difference between the current time step *t* and the time when the input spike occurs $t_{i}^{f}$ determines the trace (for ReSuMe) or kernel value (for PSD and SPAN).

**Figure 6:**
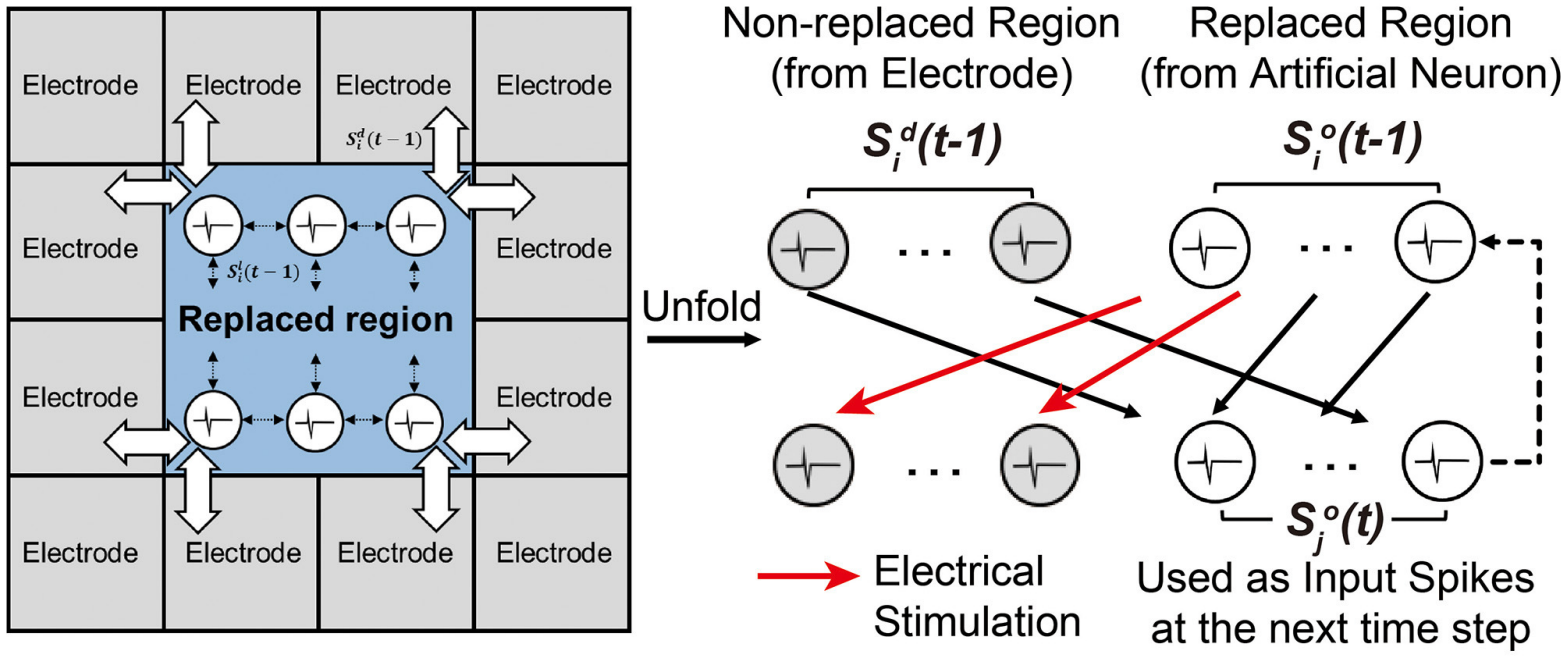
Experimental setup for replacing a sub-region of a biological neural circuit with an SNN model.

After training the SNN through a set of training sessions, it becomes possible to replace some biological neurons with trained artificial neurons and their synaptic weights. In Figure 6, white neurons are artificial neurons in the replaced region, and gray neurons are biological neurons in the non-replaced region. Note that every neuron is connected to all neurons except itself (*fully-connected*). Spikes from biological neurons in the non-replaced region $S_{i}^{d}\left(t-1\right)$ are measured spikes by the MEA. Spikes from artificial neurons in the replaced region $S_{i}^{o}\left(t-1\right)$ are computed by the SNN model. All synaptic connections to or from a neuron in the replaced region are modeled with the trained weights. Generated spikes at the artificial neurons in the replaced region $S_{i}^{o}\left(t-1\right)$ are assumed to propagate to the non-replaced region by electrical stimulation, as demonstrated by many prior works (Bruzzone et al., 2015; Chou et al., 2015; Buccelli et al., 2019).

## 3. Results

### 3.1. Experimental results on biological neural circuit

#### 3.1.1. Spike prediction accuracy with SNN (no replacement)

**Figure 7:**
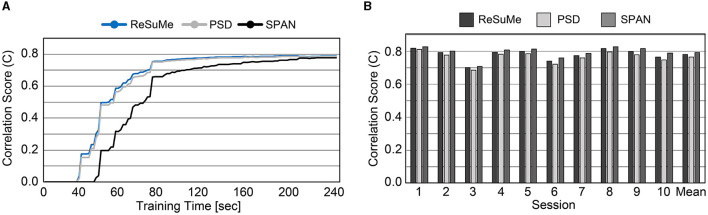
**(A)** Convergence speed of each supervised learning rule on the cell culture data (session 1). **(B)** Spike prediction accuracy of various supervised learning rules at each training session.

**Figure 8:**
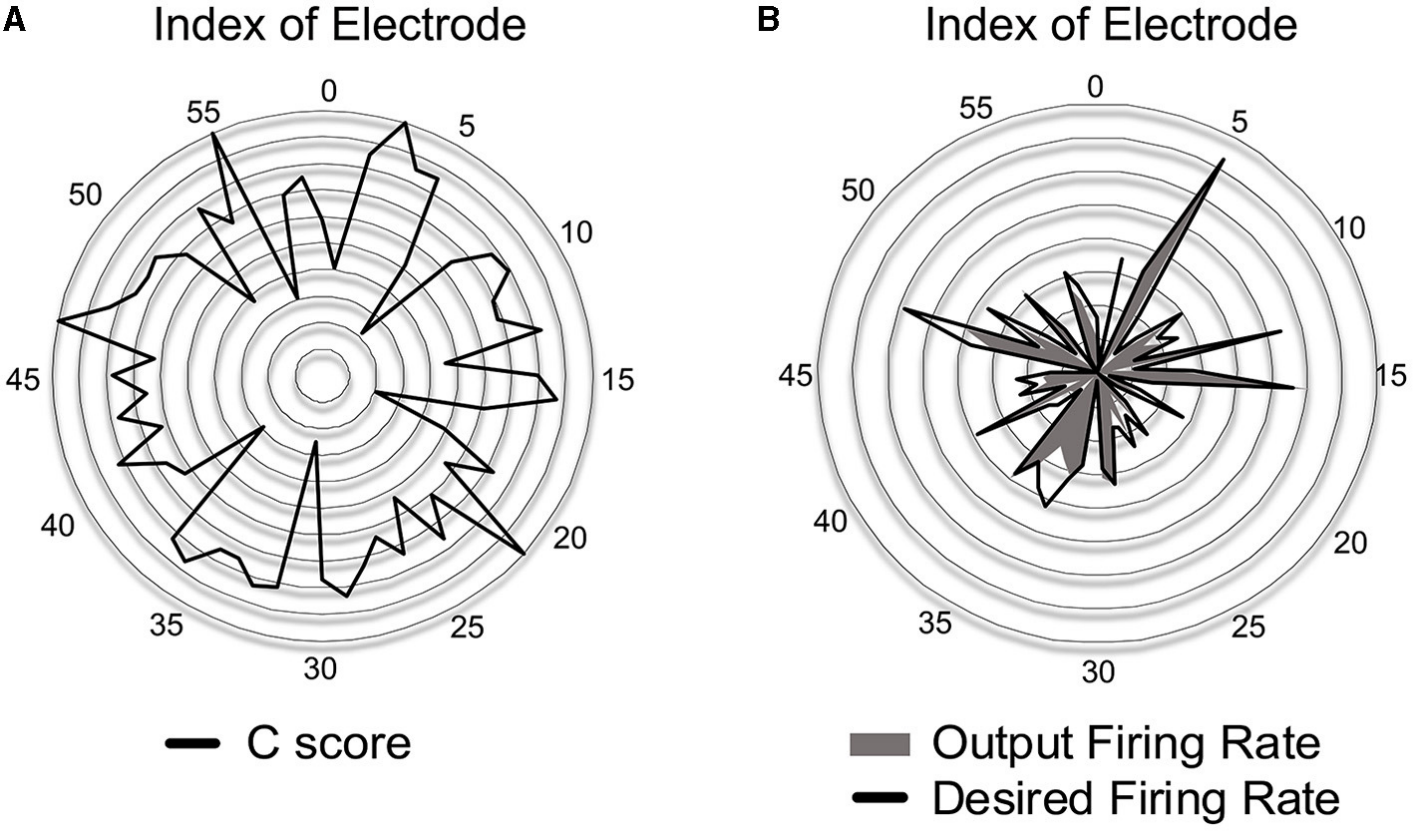
**(A)** Accuracy of spike-timing learning in terms of the correlation score *C* at each electrode when trained by ReSuMe. **(B)** Comparison of firing rate between the desired and output spike trains when trained by ReSuMe.

Prior to evaluating SNN accuracy in replacing a biological neural circuit, we need to validate the accuracy of the trained SNN model in mimicking the behavior of the biological neural circuit. The experimental setup is identical to Figure 5, and the accuracy is measured by the correlation-based score (Equation 8). In this section, we assume fully-connected SNN models and the supervised learning rules are directly used without evaluating the functional connectivity by the STDP rule. To check the SNN trainability on each session (10 min), we trained the weights for 8 min. Then, we validated the spike prediction accuracy using the MEA data of the remaining 2 min using the first 10 sessions. We compared the convergence speed of training between different supervised learning rules. As shown in Figure 7A, all three spike-timing learning rules converge to a spike correlation of 0.8 with more than 200 s of training on session 1. The convergence speed of SPAN is lower than the other learning rules, but the final accuracy is slightly higher. Figure 7B shows the validation accuracy at each session using the three learning rules. The mean accuracy for all sessions was about 76–79% with variation of 3.6–3.8%. In Figure 8, the correlation score and firing rate per neuron are shown when ReSuMe is used for training. The correlation score is relatively low for some neurons that fire little due to the lack of target spikes to be trained. The firing rate of the actual spike train $S_{j}^{d}\left(t\right)$ is shown in Figure 8B as a reference (*black solid line*). The firing rate of the output spike train $S_{j}^{o}\left(t\right)$ from the SNN has a high correlation of 0.91 with $S_{j}^{d}\left(t\right)$.

#### 3.1.2. Accuracy with sub-region replacement

**Figure 9:**
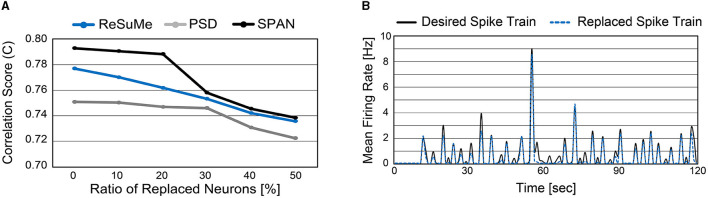
**(A)** Spike prediction accuracy when a sub-region of biological neurons is replaced. The ratio of replaced neurons is varied from 0 to 50%. **(B)** Mean firing rate of replaced neurons and the associated biological neurons (measured from electrodes). Neurons from indices 15–44 were replaced with artificial neurons (50% replacement). The ReSuMe learning rule trains the SNN model.

So far, we demonstrated the accuracy of spike prediction with the well-known supervised learning rules on our cell culture data. Note that the supervised learning rules have been only tested on synthetic data previously (Ponulak and Kasiński, 2010; Mohemmed et al., 2013; Yu et al., 2013), while this work applies these learning rules on the *in vitro* cultured network. As our main objective is to replace a sub-region of the biological neural circuit with the SNN, we replace some biological neurons with artificial neurons from now on (i.e., the same experimental setup shown in Figure 6). To do so, we trained the SNN model for 15 sessions and validated the spike prediction accuracy using the remaining five sessions with a sub-region being replaced. We varied the ratio of replaced neurons from 0 to 50% and observed the correlation score to measure the spike prediction accuracy (Figure 9A). As expected, the more neurons are replaced, the higher the prediction error. Since the biological neural circuit is recurrent, incorrect spike prediction leads to errors in subsequent predictions. As shown in Figure 9A, spike prediction with SPAN provides the highest accuracy at all replacement ratios. Even when 50% of biological neurons are replaced with artificial neurons, the correlation score becomes higher than 0.72 for all learning rules.

**Figure 10:**
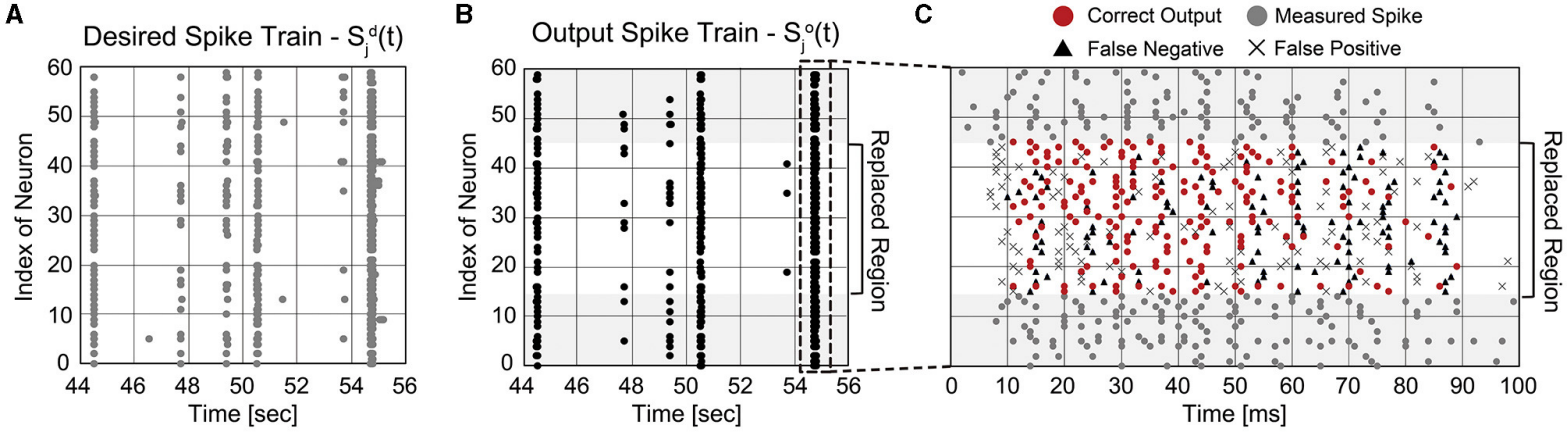
Temporal raster plots of **(A)** the original spike train and **(B)** estimated spike train during several spike bursts (12 s long in total). **(C)** The detailed raster plot view at the last spike burst event (from 54.7 to 54.8 s).

It is important not only to improve the correlation score but also to estimate the burst pattern accurately. Figure 9B presents the mean firing rate of replaced neurons when 50% of biological neurons are replaced. For the comparison, the actual mean firing rate of biological neurons is also presented (*black solid line*). The replaced spike train generates spike bursts at similar times to the desired spike train, which is crucial to understanding and mimicking neuronal behaviors (Zeldenrust et al., 2018). A spike burst can be defined as a set of spikes lasting up to 100 ms that occur together from multiple neurons. Figures 10A, B present raster plots of the original spike train [$S_{j}^{d}\left(t\right)$] and the estimated spike train [$S_{j}^{o}\left(t\right)$], respectively, during several spike bursts. The neurons from indices 15–44 were replaced by artificial neurons of the SNN model trained by ReSuMe. By comparing Figures 10A, B, it is clear that burst patterns are accurately predicted. Figure 10C shows the magnified raster plot view near *t* = 55 s where the last spike burst happens.

### 3.2. Real-time replacement of a biological neural circuit with the proposed SA-STL rule

In order to replace biological neurons in real-time, a sub-network consisting of artificial neurons needs to be computed within 1 ms (*short latency*). In addition, since the actual replacement (*in vivo*) will be made with an implantable chip, hardware needs to be designed with the minimum resources (*small form factor*). Therefore, we apply the SA-STL proposed in Section 2.2 to reduce the number of connections within the SNN model to minimize the required hardware resources and computations for the replacement. Since the SA-STL rule makes the SNN model sparse, we designed an SNN hardware capable of processing sparse computations with pipelined execution for real-time processing. Then, the spike prediction accuracy is measured by running the replaced region, a part of the trained SNN model, on the hardware accelerator.

#### 3.2.1. Hardware implementation of sparse SNNs

**Figure 11:**
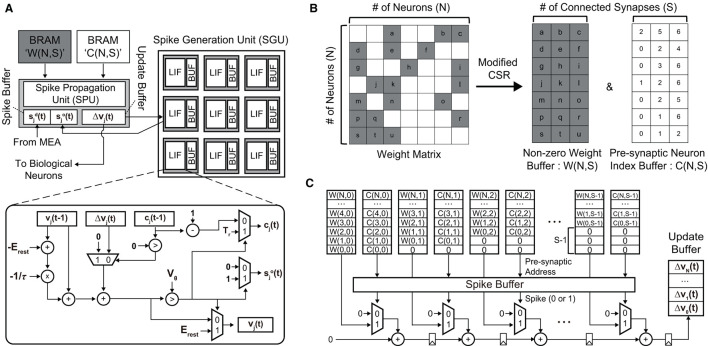
**(A)** The overall architecture of the proposed SNN hardware for the real-time replacement of a sub-region of a neural circuit. **(B)** The modified compressed sparse row (CSR) format is used to minimize the required memory footprint. **(C)** The spike propagation unit (SPU) that pipelines the accumulation path for the real-time processing (i.e., < 1 ms latency).

To demonstrate the real-time processing and analyze the required hardware resources, we implemented our SNN hardware on a small FPGA, i.e., Xilinx PYNQ-Z2 (ZYNQ XC7Z020). The clock frequency is set to 50 MHz to keep the power consumption of the SNN hardware low. To process an SNN model, we need to place processing units for (i) updating membrane potentials of neurons and (ii) propagating spikes via weighted synapses. These processing units are depicted in Figure 11A. The spike generation unit (SGU) updates the membrane potential *v*_*j*_(*t*) of a post-synaptic neuron *j* and generates a spike $s_{j}^{o}\left(t\right)$ when the potential exceeds the threshold *V*_θ_. The potential increase/decrease Δ*v*_*j*_(*t*) is computed by the spike propagation unit (SPU), which will be explained shortly. When the neuron fires, i.e., $s_{j}^{o}\left(t\right)=1$, then counter value *c*_*j*_(*t*) is set to *T*_*r*_ (refractory period). During the refractory period, the membrane potential is not updated [Δ*v*_*j*_(*t*) is neglected]. The SGU has the same number of LIF units as the number of replaced neurons, where LIF units operate in parallel for higher throughput.

To compute the potential update vector $\Delta \vec{v}\left(t\right)$, we need to perform matrix-vector multiplication between the synaptic weight matrix **W**∈ℝ^*N*×*N*^ and the pre-synaptic spike vector $\vec{s}\left(t-1\right)=\left\{\vec{s^{d}}\left(t-1\right),\vec{s^{o}}\left(t-1\right)\right\}$. Here, $\vec{s^{d}}$ is the spike vector of non-replaced neurons measured by the MEA, and $\vec{s^{o}}$ is the spike vector of replaced neurons computed by the SNN model. Our SA-STL rule cuts less critical synapses by running the STDP rule at each training session, which makes **W** sparse, as shown in Figure 11B. We convert **W** to a modified CSR format to store the sparse weight matrix in a small memory block. Since we strictly limit the number of synapses per post-synaptic neuron to *S*, i.e., *N*_*syn*_ in Algorithm 1, the number of non-zero weights per row remains the same. In the SPU shown in Figure 11C, the sparse **W** stored in the modified CSR format is used to perform weighted spike accumulations, i.e., $\Delta v_{j}\left(t\right)=\overset{S-1}{\underset{k=0}{\sum }}W\left(j,k\right)\cdot s_{i}\left(t-1\right)$ where *i* = *C*(*j, k*). An element in the pre-synaptic neuron index buffer, i.e., *C*(*j, k*), points to the pre-synaptic spike *s*_*i*_(*t*−1) in the spike buffer connected to the post-synaptic neuron *j*. Only when *s*_*i*_(*t*−1) = 1, the synaptic weight *W*(*j, k*) is added. For real-time processing, the accumulation path is pipelined to improve the throughput.

**Figure 12:**
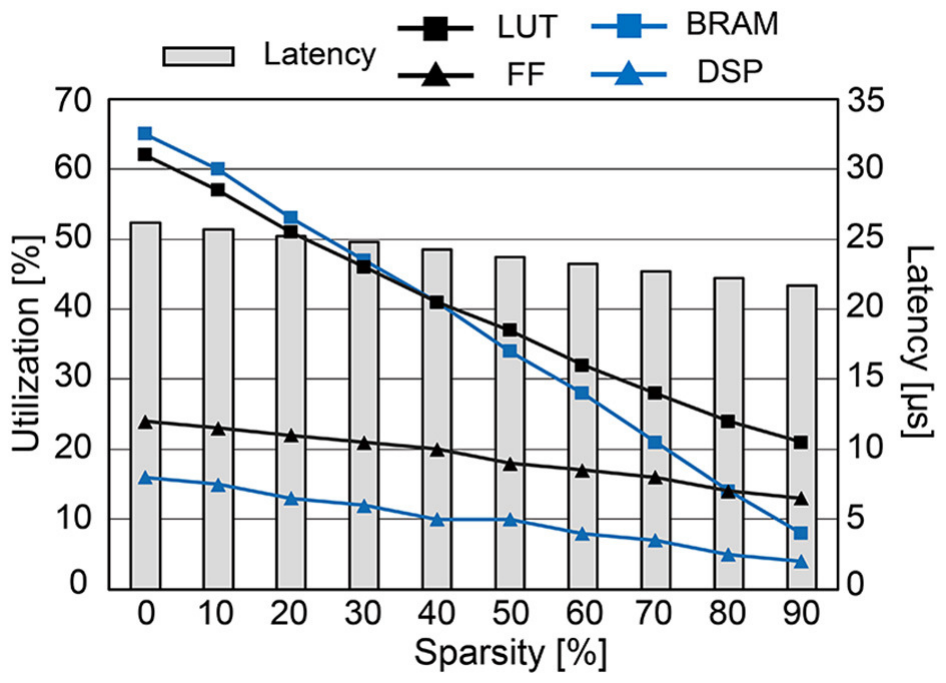
The hardware utilization and compute latency of the proposed SNN hardware for each timestep (1 ms) at various sparsity levels.

Figure 12 presents the hardware utilization and compute latency of our SNN hardware at various sparsity levels. Increasing the sparsity of **W** reduces the required hardware resources. Look-up tables (LUTs), flip-flops (FFs), and digital signal processing units (DSPs) are used to realize the datapath and its related control signals. Block RAMs (BRAMs) are memory blocks that store the weight matrix **W**, potential update vector $\Delta \vec{v}\left(t\right)$, and spike vector $\vec{s}\left(t\right)$. The size of BRAMs decreases linearly with high sparsity due to the reduction in data size by storing the weight matrix in the modified CSR format. If we do not utilize the CSR format, BRAM usage will increase since zero-valued weights also need to be stored. Despite the overhead of storing an index buffer in addition to the weight matrix, our modified CSR format reduces BRAM usage by 2.5 × compared to the case without using CSR at 80% sparsity. Reducing the required hardware resources is crucial to designing a small SNN chip that consumes less power. Moreover, the computation latency is kept below 27 μs, i.e., far < 1 ms (*real-time execution*), in all cases owing to the pipeline execution and parallel computation. Compared to the hardware without pipelined execution, it was possible to reduce the latency by 4.2 × (when 80% sparsity) or 14.3 × (when 0% sparsity). The power consumption tends to decrease when the sparsity increases through SA-STL learning. As presented in Table 1, the dynamic power of all-to-all connected SNN without SA-STL is estimated to be 151 mW. On the other hand, with SA-STL, the dynamic power at 80% sparsity becomes 114 mW, which is about 25% less power than the fully-connected case.

**Table 1:** Hardware performance comparison between sparse SNN (80% sparsity with SA-STL) and fully-connected SNN.

**Zynq7020 (Clock: 50 MHZ)**	**BRAM utilization (%)**	**LUT utilization (%)**	**Dynamic power (mW)**	**Latency (μs)**
SA-STL (80% sparsity)	14	25	114	22.24
All-to-all connections	65	62	151	26.16

#### 3.2.2. Accuracy of real-time replacement with SA-STL rule

**Figure 13:**
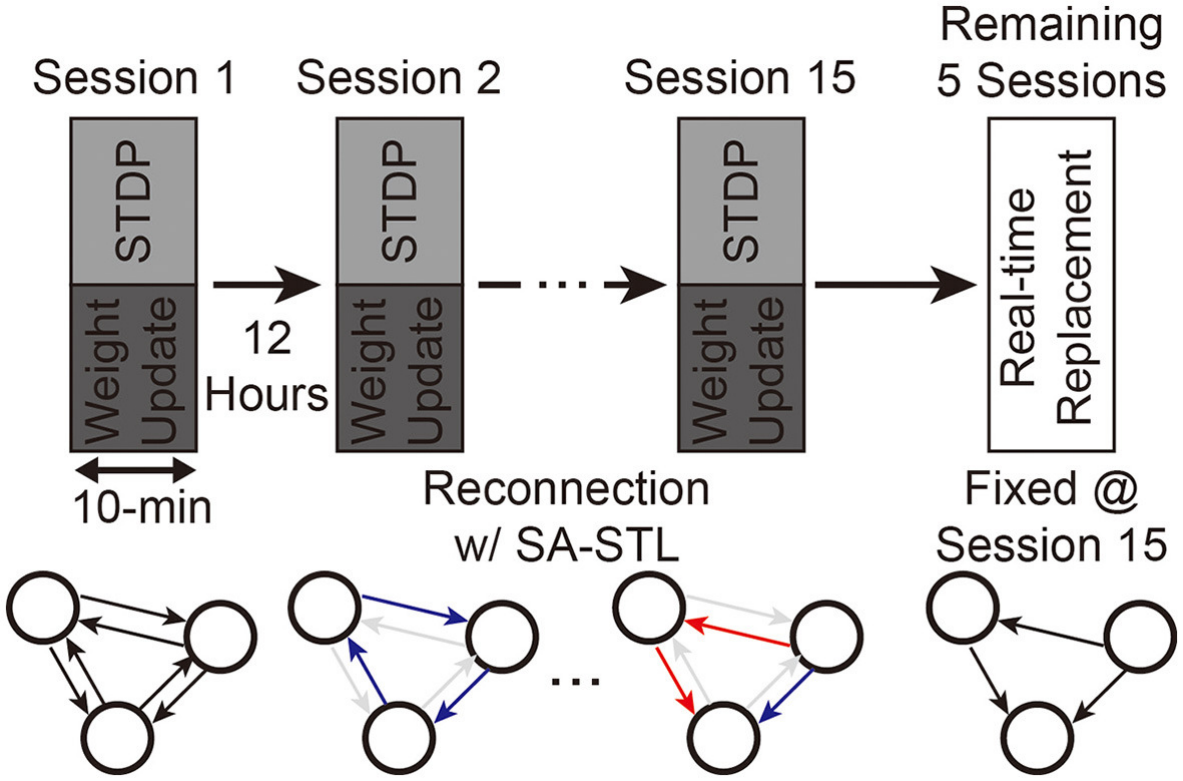
Experimental process of training an SNN model for the sub-region replacement and deploying the trained model on the SNN hardware for the real-time replacement.

With the support of our SNN hardware, we can generate spikes at the replaced region with artificial neurons in real-time. Since we can minimize the required hardware resources by increasing the sparsity of **W**, we trained the SNN model at various sparsity levels using the proposed SA-STL rule. As explained in Section 3.1, we use the first 15 sessions as a training dataset, and the remaining five sessions are used to validate spike predictions with sub-region replacement. The experimental process is illustrated in Figure 13. Synapses to be connected at the next training session are selected by the STDP rule at the previous training session.

**Figure 14:**
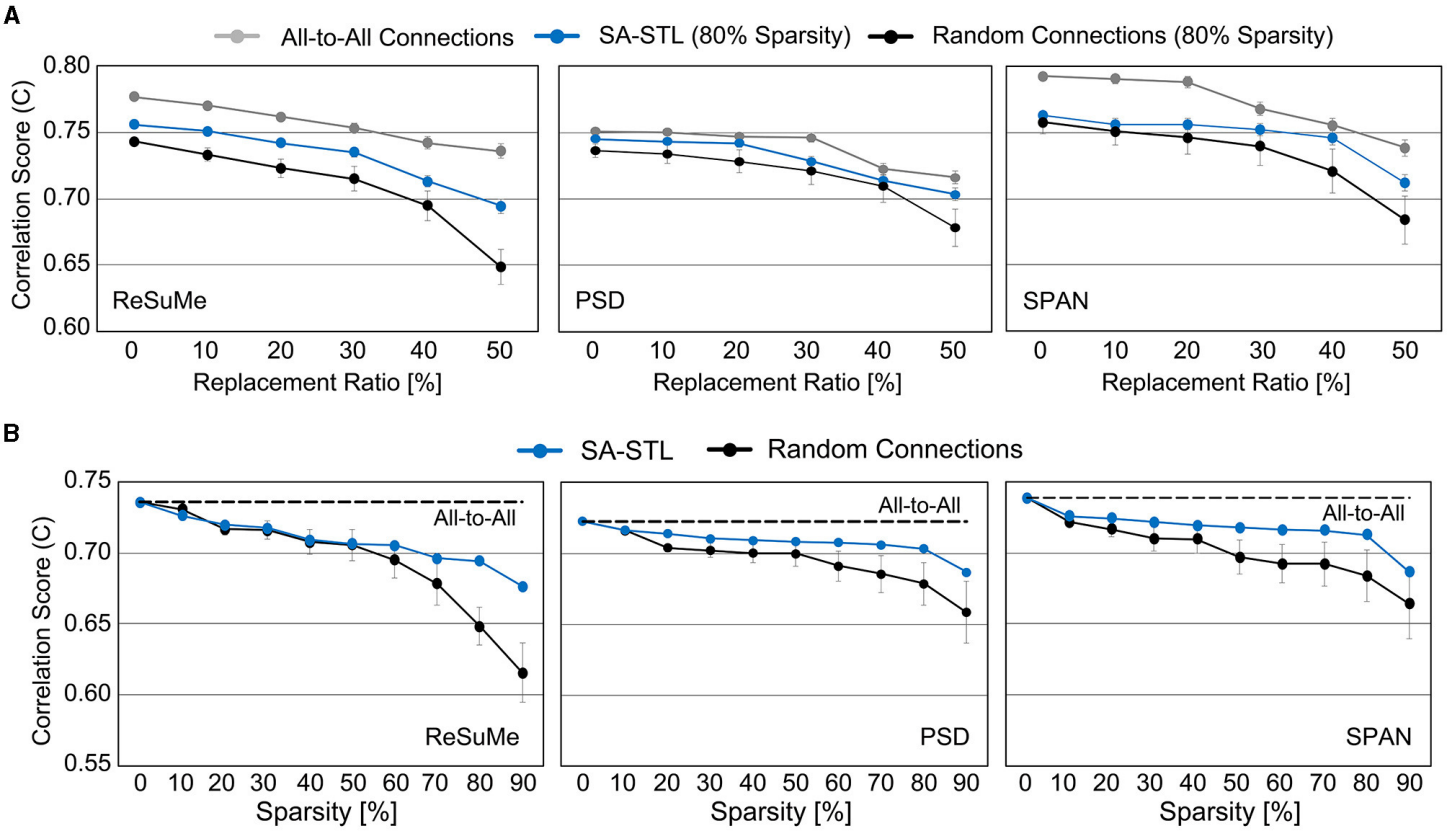
**(A)** Comparison of spike prediction accuracy between SNNs using all-to-all connections, SA-STL, and random connections at various replacement ratios. The sparsity level for the SNN with SA-STL or random connections is selected as 80%. **(B)** Comparison of spike prediction accuracy between SNNs using SA-STL and random connections at various sparsity levels. In this experiment, 50% of biological neurons were replaced with artificial neurons. The cutoff electrodes were randomly determined and experimented with 20 trials. Due to a high variation in experiments with random connections, we conducted 20 trials to present average values.

Weak synapses are removed from the connectivity list at each training session, and stronger synapses are newly connected. To stabilize the training, we initialized the weights of newly connected synapses to the mean weight value of the remaining synapses. After 15 training sessions, the synapses are fixed, and their weights are programmed into the SNN hardware for real-time replacement. Figure 14A presents the spike prediction accuracy at various replacement ratios from 0 to 50%. For the hardware efficiency, only 20% of synapses are connected when training the SNN model, i.e., 12 pre-synaptic neurons are connected to a post-synaptic neuron. Obviously, allowing all-to-all connections, i.e., 0% sparsity, achieves the highest accuracy with significant hardware overhead. By randomly selecting a part of synapses to be connected for better hardware efficiency, however, the accuracy significantly degrades by 4.77, 2.31, and 3.76% on average when trained with the ReSuMe, PSD, and SPAN rule, respectively. Using the proposed SA-STL rule, we can improve the accuracy by 2.32, 1.43, and 1.46% on average using the ReSuMe, PSD, and SPAN rules, respectively. Therefore, the SA-STL rule can be used as a stable learning method that keeps the accuracy as high as possible with a limited number of synapses. With a larger sub-region replaced, e.g., 50%, the average spike prediction accuracy degrades by 2.88%. Regarding the learning rule, SPAN provides the best accuracy, while PSD shows the lowest accuracy.

To see how the accuracy changes with respect to the sparsity level of an SNN model, we varied the sparsity level from 0 to 90%. Similar to the results shown in Figure 14B, the prediction accuracy with SPAN was the highest among three learning rules. The ReSuMe rule's accuracy drops faster than the other two, implying that it is less suitable for training sparse SNNs for precise spike-timing learning. At all sparsity levels, sparse SNNs trained with the proposed SA-STL rule perform better than the randomly connected SNNs. Compared to the SNN with the proposed SA-STL, the variance of the prediction accuracy of a randomly connected SNN was larger, and the mean accuracy was much lower. To keep the correlation score higher than 0.70, we need to keep the sparsity of an SNN model at 80% or lower.

**Figure 15:**
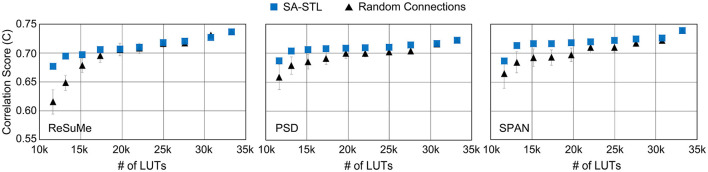
The spike prediction accuracy at a given number of LUTs in the FPGA (i.e., Xilinx PYNQ-Z2). The sparsity level of an SNN model is determined by the given number of LUTs. In this experiment, 50% of biological neurons were replaced with artificial neurons.

To see the impact of the hardware budget on the spike prediction accuracy, we constrained the number of LUTs and analyzed the accuracy. As shown in Figure 15, the reduction in prediction accuracy is minimized by using the proposed SA-STL rule for training a sparse SNN model. With the support of SA-STL, the required number of LUTs reduces by 2.5 × with only 2.3% accuracy loss using the SPAN rule. For more practical use cases, we may need to implement hardware for an SNN model with a greater number of neurons. The number of computations involved in processing the SNN model is proportional to *N*^2^ where *N* is the number of neurons in the network. Therefore, our SA-STL rule becomes more effective when we scale the size of the SNN model that replaces the biological neural circuit.

## 4. Discussions

In this paper, we presented a novel learning algorithm, SA-STL, to efficiently remove synapses in an SNN model that replaces a sub-region of a biological neural circuit. The proposed SA-STL rule dynamically selects synapses that have more relevance to predicting spike timings of the target neural circuit. Then, the hardware prototype was designed on a small FPGA to reproduce spikes at the replaced region in real-time. To demonstrate the effectiveness of our software-hardware co-design approach, we collected neural recording data to conduct more realistic experiments. This work can be seen as an initial step for multidisciplinary research to replace a brain function with SNN hardware. Compared to the fully-connected SNN, our sparse SNN hardware could infer the spikes of the replaced sub-region in 22 μs with 2.5 × fewer hardware resources. It will have a more significant impact when we replace the brain functionality of a larger region in real-time using an implantable chip.

Based on this initial set of experiments, our future work is to implement a closed-loop system where real-time spike communication happens between the main neural circuit (BNN) and the replaced SNN via electrical stimulation. This can be done by developing a precise electrical stimulation system that stimulates biological neurons connected to an SNN. Currently, our work assumes that such a stimulation system is available, and we allow inferred/measured spikes to convey data without any loss across BNN-SNN boundaries. Developing a precise electrical stimulation system along with low-impedance electrodes is one of our future works and is a fundamental challenge for repairing damaged neural circuits. Another challenge in processing spike trains in real-time is spike sorting, a process to identify the location of a neuron that has generated the spike at each electrode. Therefore, hardware for real-time spike classification across a large number of electrodes becomes another future research direction. Despite these limitations, this work presents an essential step toward real-time computation for neural prosthetics. Beyond the cultured hippocampus, we could replace a neural function at an impaired sub-region of the human brain with SNNs.

## Data availability statement

The raw data supporting the conclusions of this article will be made available by the authors, without undue reservation.

## Ethics statement

The animal study was reviewed and approved by IACUC of DGIST (DGIST-IACUC-21041903-0002). Written informed consent was obtained from the owners for the participation of their animals in this study.

## Author contributions

SH: algorithms and hardware implementation. YH, DK, and JunhL: cell culture and data collection. HC: experimental setting for cell culturing. JungL: advice on the signal acquisition. HK: advice on cell culture process. JK: advice on SNN hardware implementation. All authors contributed to the article and approved the submitted version.
