# Supplementary material for: ReplaceNet: real-time replacement of a biological neural circuit with a hardware-assisted spiking neural network
Source: Front Neurosci. 2023 Aug 10;17:1161592. doi: 10.3389/fnins.2023.1161592 (PMC10448768; doi:10.3389/fnins.2023.1161592)
Supplement: Supplementary file 1 [file Data_Sheet_1.PDF]

Supplementary Material

ReplaceNet: Real-time Replacement of a Biological Neural Circuit with a Hardware-assisted Spiking Neural Network

1 SUPPLEMENTARY: CELL CULTURE PROTOCOLS

The details of biological experimental protocols on cell culture are as follows. Fetal rats were extracted from the 18-day pregnant Sprague Dawley rat (TP18, SD-rat) to obtain embryo hippocampus. First, a pregnant rat's abdominal cavity was opened using sterile scissors and forceps after undergoing anesthesia with carbon dioxide (CO_2) gas for at least 10 minutes. After cutting the mesometrium to remove the uterus, the uterus was transferred into a petri dish filled with fresh Hanks' balanced salt solution (HBSS, 10x, 21103-049, Gibco). After removing the embryonic sacs from the uterus, we extracted the brain of the embryo by taking off the scalp and cranial bone along the sagittal and coronal sutures. Cerebral hemispheres were divided right down the middle into the right and left hemispheres. The hippocampus was cut out from each hemisphere and stored in the HBSS. The hippocampal neurons were mechanically dissociated using pipetting about 20 times, blending thoroughly. By centrifuging (1,000 rpm for 2 minutes), we carefully removed the supernatant and moved it to a plating culture medium. The culture maintenance medium was prepared as a mixture of Neurobasal medium with 2% of B-27 (17504-044, Gibco), 2 mM of Glutamax (35050-061, Gibco), and 1% Penicillin-Streptomycine (15140-122, Gibco). For the initial plating medium, 12.5 µM L-glutamic acid (G8415, Sigma-Aldrich) was added to the maintenance medium. Filtered cell suspension through a strainer (Falcon 40 µm pores, Corning, USA) was seeded onto the prepared microelectrode array chips. Before the cell seeding, the MEAs were cleaned by immersing them in deionized water for at least 12 hours. For the adhesion of cultured neurons, the surface of the MEAs was coated with 0.05 mg/ml of poly-D-lysine (a3890401, Gibco) for at least 12 hours in a humidified incubator (37°C, 5% CO_2). After the surface coating, the MEA was rinsed multiple times with sterilized water and 70% ethanol. The hippocampal neurons were cultured on the MEAs at an approximate density of 1,000 $cells/mm^2$. After the neuronal cell seeding, the MEAs were incubated for at least 30 min to allow the seeded cells to adhere strongly to the surface of the MEAs. After the 30-min incubation, the MEAs were filled with the prepared plating medium of 1$\sim$2 ml. After that, the cultured MEAs were incubated (37°C, 5% CO_2), and half of the medium was exchanged with a fresh maintenance medium twice a week. Spikes from the recorded biological neuronal network signals were detected using a threshold method (-5.5x of RMS noise) from commercial software (Multi Channel Analyzer, Multi-Channel Systems MCS GmbH), and the time stamps of the detected spikes for each channel were exported for the input of the SNN.
